# Red-Koji Fermented Red Ginseng Ameliorates High Fat Diet-Induced Metabolic Disorders in Mice

**DOI:** 10.3390/nu5114316

**Published:** 2013-10-30

**Authors:** Chang Man Kim, Seong Joon Yi, Il Je Cho, Sae Kwang Ku

**Affiliations:** 1 Department of Science Education, College of Education, Daegu University, Gyeongsan, Gyeongsangbuk-do 712-714, Korea; E-Mail: cmkim250@hanmail.net; 2 Department of Anatomy, College of Veterinary Medicine, Kyungpook National University, Daegu 702-701, Korea; E-Mail: sjyi@knu.ac.kr; 3 MRC-GHF, College of Korean Medicine, Daegu Haany University, Gyeongsan, Gyeongsangbuk-do 712-715, Korea

**Keywords:** fermentation, high fat diet, metabolic disorders, obesity, red-koji, red ginseng

## Abstract

Fermentation of medicinal herbs improves their pharmacological efficacy. In this study, we investigated the effects of red-koji fermented red ginseng (fRG) on high-fat diet (HFD)-mediated metabolic disorders, and those effects were compared to those of non-fermented red ginseng (RG). fRG (500, 250 or 125 mg/kg), RG (250 mg/kg), simvastatin (10 mg/kg), silymarin (100 mg/kg) and metformin (250 mg/kg) were orally administered from 1 week after initiation of HFD supply for 84 days. The diameter of adipocytes in periovarian and abdominal fat pads and the thickness of the abdominal fat were significantly decreased by fRG treatment, while HFD-mediated weight gain was partly alleviated by fRG in a dose-dependent manner. Moreover, biochemical and histomorphometrical analyses clearly indicated that fRG significantly inhibited HFD-induced metabolic disorders such as hyperglycemia, hyperlipidemia, hepatopathy and nephropathy in a dose-dependent manner. More favorable pharmacological effects on HFD-mediated metabolic disorders were also observed with fRG compared to an equal dose of RG. This finding provides direct evidence that the pharmacological activities of RG were enhanced by red-koji fermentation, and fRG could be a neutraceutical resource for the alleviation of obesity-mediated metabolic disorders.

## 1. Introduction

The worldwide incidence of obesity continues to escalate, despite increased awareness and global efforts to understand and confront its origins. Obesity is caused by an energy imbalance accompanied by complicating genetic and socio-economic factors [1]. According to the report from WHO, obesity is the fifth leading risk for global deaths and at least 2.8 million adults die each year as a result of being overweight or obese. Obesity is consequently regarded as one of the major risk factors to increase morbidity and mortality of metabolic syndromes such as diabetes mellitus, hyperlipidemia, hypertension, and cardiovascular disease [2,3].

Excessive intake of fatty acids leads to an accumulation of triglyceride in many tissues, particularly in the fat tissue, in which lipolysis is increased. The increased levels of fatty acids in the circulatory system facilitate the uptake of fatty acids in peripheral tissue through the induction of fatty acid binding and transport proteins (e.g., FABP and CD36) [4]. The exaggerated availability and deposition of free fatty acids induce lipotoxicity and insulin resistance in peripheral tissues [5,6]. Furthermore, a high concentration of free fatty acids contributes to triglyceride accumulation in the liver. Prolonged and repeated accumulation of triglyceride in liver increases the possibility of inflammation, hepatocellular necrosis and fibrosis [7]. In addition, obesity aggravates the course of many primary renal diseases such as glomerulonephritis and also impairs renal function [5]. Although many kinds of animal models are used to develop new drugs for obesity-mediated metabolic disorders, most of these models exhibit serious obesity and hyperglycemia and are only suitable for investigating the treatment of established diabetes [8]. Obesity in mice is developed by feeding them a high-fat diet (HFD), and the obese mice have the characteristics of hyperglycemia, insulin resistance, hepatic steatosis, mild diabetic nephropathy and hyperlipidemia [9,10,11]. Therefore, an animal model using HFD is appropriate for developing preventive agents for obesity-mediated metabolic disorders [9].

However, currently available pharmacological agents for obesity-mediated metabolic disorders have a number of limitations, such as various adverse effects and high rates of secondary failure [12]. Patients and healthcare professions have therefore been interested in complementary and alternative approaches, including the use of medicinal herbs. Ginseng (Root of *Panax ginseng* C.A. Meyer, family Araliaceae) is one of the most commonly used medicinal herbs to enhance body strength and stimulate metabolic function in Asian countries [13]. To enhance the biological and pharmacological activities of *P*. *ginseng*, red ginseng (RG) is made by steam-processing *P*. *ginseng* [14]. It has been reported that RG alleviates obesity-mediated metabolic disorders [10,15]. Moreover, fermentation of RG using a variety of edible microorganisms seems to further enhance the pharmacological efficacy of RG [16,17,18]. Red-koji prepared from *Monascus* species has traditionally been used as a medicinal food itself and/or as a fermentation source for other herbs in East Asia [19]. However, the effects of Red-koji fermented RG (fRG) on obesity-mediated metabolic disorders have not been thoroughly studied.

We hypothesized that the pharmacological effects of RG could be improved by fermentation with red-koji. Therefore, this study examined the anti-obesity, hypoglycemic, hypolipidemic, hepatoprotective and nephroprotective effects of fRG in HFD-fed mice for 84 days, and those efficacies were compared to those of non-fermented RG.

## 2. Materials and Methods

### 2.1. Animals

Animal studies were performed in accordance with the institutional guidelines of Daegu Haany University for the Care and Use of Laboratory Animals. Experimental procedures were reviewed and approved by the Ethical Committee for Animal Experimentation of the Daegu Haany University (Protocol# DHU2011-002). One-hundred and eighty female ICR mice (6 weeks old, SLC, Shizuoka, Japan) were used in this study after 7 days of acclimatization. 45%/kcal HFD and normal rodent pellet diet (ND) were supplied by Diet Research (Bethlehem, PA, USA) and Superfeed Co. (Seoul, Korea), respectively. Animals were allocated four per polycarbonate cage in a temperature (20–25 °C) and humidity (40%–45%) controlled room. The light:dark cycle was 12:12 h, and diet and water were supplied *ad libitum*. One-hundred and sixty mice were supplied HFD and 20 mice were fed ND throughout the experimental period. Out of 180 mice, 80 adapted mice on HFD were selected based on their body weights at 7 days after initiation of HFD supply, and 2 mice in each group were removed based on the standard deviation of body weight at the end of the 84-day administration period (final of 8 mice per group). A total of 72 mice were sacrificed.

### 2.2. Preparation and Administration of Test Materials

fRG and RG were prepared and provided by Ginseng Organic Co. (Seoul, Korea). Simvastatin was kindly supplied by Dong Wha Pharm. Co. (Yongin, Korea). Silymarin and metformin were purchased from Sigma (St. Louis, MO, USA) and Wako Pure Chemical (Osaka, Japan), respectively. fRG (500, 250, or 125 mg/kg) and RG (250 mg/kg) were suspended in distilled water, and orally administered to HFD-fed mice from 1 week after initiation of HFD supply for 12 weeks daily. Simvastatin (10 mg/kg) for hypolipidemic effect, silymarin (100 mg/kg) for hepatoprotective effects and metformin (250 mg/kg) for hypoglycemic and related anti-obesity effects were used as reference drugs. The three reference drugs were also directly suspended or dissolved in distilled water, and orally administered as previously described [9].

### 2.3. Measurement of Body and Organ Weights

The body weights were measured at 1 day before initiation of administration, and weekly from initial administration day to termination day using an automatic electronic balance (Precisa Instrument, Switzerland). At initiation and termination days, all experimental animals were fasted overnight (water was provided; about 12 h) to reduce any differences from feeding. At sacrifice, the weights of periovarian fat pads, liver and kidney were measured, and relative body weight was calculated using the body weight at sacrifice and the absolute organ weight as follows: (absolute organ weight/body weight at sacrifice) × 100.

### 2.4. Measurement of Mean Daily Food Consumption

150 g of diet were supplied per individual cages, and any remaining amount was measured at 24 h using an automatic electronic balance (Precisa Instrument, Switzerland). The mass consumed was then divided by the number of animals in the cage, and this was regarded as the individual mean daily food consumption (g/day/mouse). These measurements were conducted once a week during the 84-day administration period.

### 2.5. Blood Biochemistry

Blood was collected from the vena cava. All blood samples were centrifuged at 15,000 rpm for 10 min at room temperature in a clotting activated serum tube. Serum aspartate aminotransferase (AST), alanine aminotransferase (ALT), total cholesterol, triglyceride, low-density lipoprotein (LDL), blood urea nitrogen (BUN), creatinine, and glucose levels were determined using an automated blood analyzer (Hemagen Analyst; Hemagen Diagnostic, Columbia, MD, USA).

### 2.6. Histopathology

The left lateral lobes of liver, the left kidney, the left periovarian fat pads, and the dorsal abdominal fat pads attached to the *muscularis quadratus lumborum* were sampled. The tissues were fixed in 10% neutral buffered formalin. After paraffin embedding, 3 or 4 μm serial sections were prepared. Representative sections were stained with hematoxylin and eosin (H & E) for light microscopic examination. Mean diameter of periovarian and dorsal abdominal white adipocytes (μm; at least 10 white adipocytes per fat pad were considered), thickness of the dorsal abdominal fat pads (mm), mean diameter of hepatocytes (μm; at least 10 hepatocytes in restricted visual field were considered), percentage of fatty change regions in hepatic parenchyma (%/1 mm^2^ of hepatic parenchyma), mean diameter of hepatocytes (μm; at least 10 hepatocytes per each liver were considered), and mean abnormal tubules (over 20% of vacuolated tubules among 100 tubules) were calculated by histomorphometry using an automated image analysis process (DMI, Daegu, Korea). The certified histopathologist was blinded to group distribution when this analysis was made.

### 2.7. Statistical Analyses

Statistical analyses were conducted using SPSS for Windows (Release 6.1.3., SPSS Inc., Chicago, IL, USA). Multiple comparison tests for different dose groups were conducted. Variance homogeneity was examined using the Levene test. If the Levene test indicated no significant deviations from variance homogeneity, the data were analyzed by one-way ANOVA followed by least-significant differences (LSD) multi-comparison test to determine which pairs in the group comparison were significantly different. If significant deviations from variance homogeneity were observed in the Levene test, a non-parametric comparison test, the Kruskal-Wallis H test was conducted. When a significant difference was observed in the Kruskal-Wallis H test, the Mann-Whitney U-Wilcoxon Rank Sum W test was conducted to determine the specific pairs in the group comparison that were significantly different. A *p* value less than 0.05 was considered significant.

## 3. Results

### 3.1. Effect of fRG on HFD-Induced Obesity

**Figure 1:**
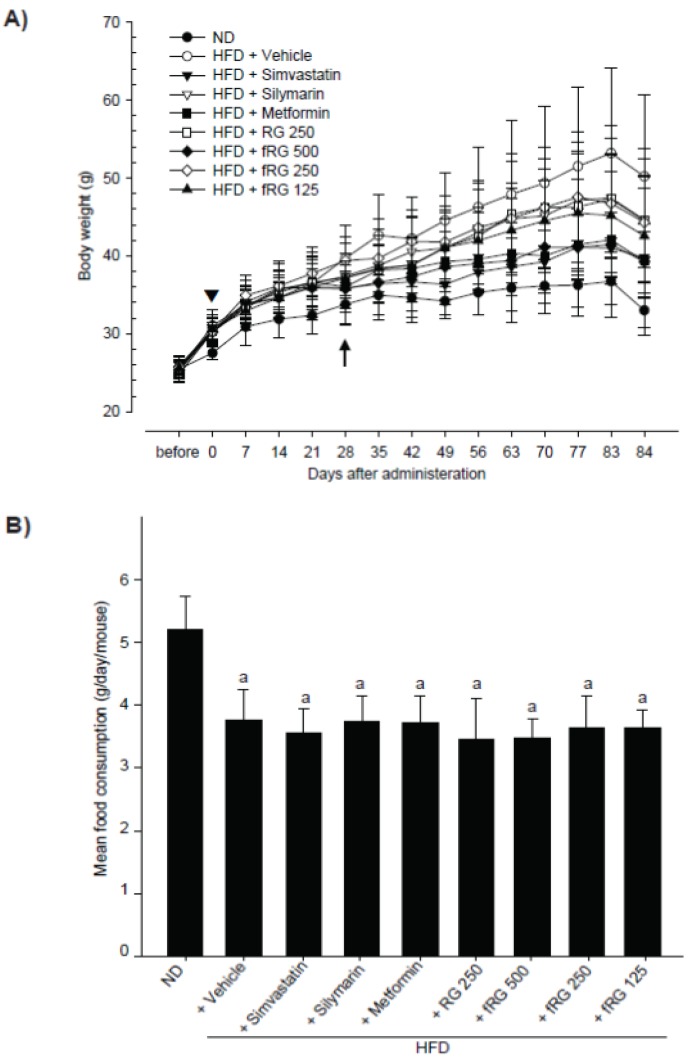
Effect of red-koji fermented red ginseng (fRG) on body weight and food consumption. Mice shown regular body weight increases in HFD-treated groups were selected in adaptation period, and treated HFD with simvastatin (10 mg/kg), silymarin (100 mg/kg), metformin (250 mg/kg), RG (250 mg/kg) or fRG (500, 250 or 125 mg/kg) for 84 days. Body weight changes (**A**) and mean daily food consumption (**B**) during experimental period were calculated as described in material and methods section. All values were expressed mean ± S.D. of eight mice (Significant as compared with normal diet–fed mice, ^a^ *p* <0.01; ND, normal diet; HFD, high fat diet; RG, red ginseng).

To investigate the therapeutic potential of fRG on HFD-mediated metabolic disorders, we first selected mice only showing body weight increases compared with normal diet-fed mice during the 1-week adaptation period. As shown in Figure 1, all HFD-fed mice exhibited significant increases in body weight (Arrow-head in Figure 1A and Table 1). A total of 80 adapted mice were divided into 8 groups and were continuously fed HFD with fRG (125, 250, or 500 mg/kg), RG (250 mg/kg) or three reference drugs (silymarin (100 mg/kg), simvastatin (10 mg/kg), metformin (250 mg/kg)) for 84 days. The body weight gain during the 84-day of HFD administration period was also significantly increased compared with that in ND-fed mice (Table 1). Although the significances decreases were restricted to certain administration times, the body weights in fRG (500 mg/kg)-, simvastatin-, and metformin-administrated groups were significantly decreased compared with HFD control from 28 days after start of administration (*p <* 0.01 or *p <* 0.05; arrow in Figure 1A). In addition, significant (*p <* 0.05) decreases of body weight gains during the 84-day administration period were only detected in simvastatin- and metformin-treated groups compared with HFD control. Although fRG prevented the weight gain in a dose-dependent manner, this effect was not statistically significant. Non-significant but marked decreases of body weight gains were also observed in silymarin- and RG-treated groups (Table 1). The body weight gains during the 84-day administration period in HFD control were changed by 250.00% compared with ND-fed control, and by −51.34%, −24.98%, −53.56%, −25.64%, −52.58%, −26.88% and −37.44% in simvastatin, silymarin, metformin, RG, fRG 500, 250 and 125 mg/kg administered groups compared with HFD control, respectively. Although significant (*p <* 0.01) decreases in mean daily food consumption were detected in all HFD-fed mice, the food consumption was not changed among all tested groups (Figure 1B).

**Figure 2:**
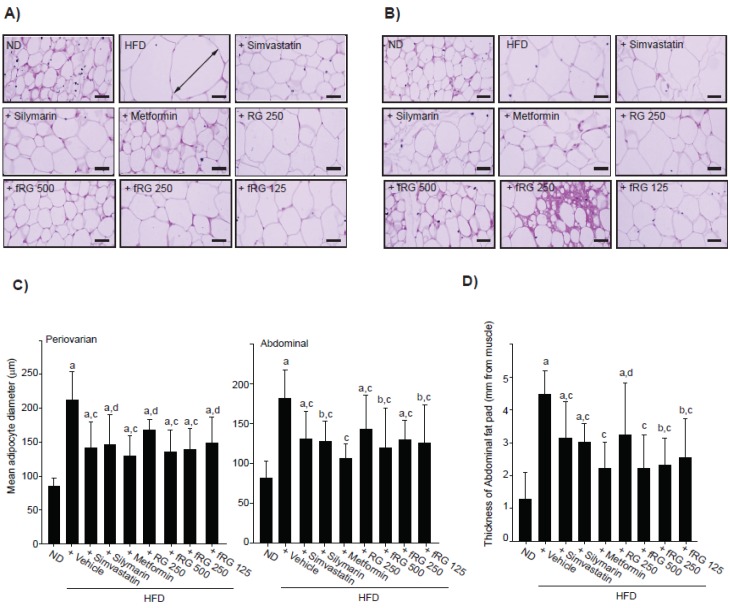
Histomorphometrical changes of the fat pad in fRG-treated mice. The representative histological images of fat from periovarian (**A**) or abdominal (**B**) tissues were stained with H & E. The arrow indicates the diameter of adipocytes measured (Scale bars = 80 μm). The mean diameter of at least 10 periovarian or abdominal white adipocytes (**C**), and the thickness of the fat pads in abdominal cavity (**D**) were measured using automated image analysis process. All values were expressed mean ± S.D. of eight mice (Significant as compared with ND-fed mice, ^a^ *p* < 0.01; ^b^ *p* < 0.05; significant as compared with HFD-fed mice, ^c^ *p* < 0.01; ^d^ *p* < 0.05).

Next, we examined the effect of fRG on HFD-induced obesity. Significant (*p <* 0.01) increases of periovarian fat pad weights were detected in HFD control compared with the ND-fed group (Table 2). However, those were reduced by 500 or 250 mg/kg fRG treatment. More favorable inhibition of the periovarian fat pad weight was detected with fRG treatment than with an equal dose of RG (Table 2). Histomorphometrical analyses of fat pads revealed that the mean diameters of periovarian adipocytes (Figure 2A,C) and abdominal adipocytes (Figure 2B,C) and the thickness of the abdominal fat pad (Figure 2D) were all increased by HFD supplementation. In contrast, three different dosages of fRG significantly reduced the mean diameters of periovarian adipocytes and abdominal adipocytes and the thickness of the abdominal fat pad. Furthermore, 250 mg/kg fRG more favorably suppressed the hypertrophy of the fat pad than an equal dose of RG.

**Table 1:** Body weight gains after 84 days of continuous oral treatment of fRG in HFD-fed mice.

Groups	Body weights (g):		Body weight gains during (g):	
	At start of administration	At a termination	Adapt period (7 days)	Administration period (84 days)
ND	27.54 ± 0.88	33.01 ± 2.28	2.04 ± 0.63	5.48 ± 1.58
HFD	31.04 ± 2.12 ^a^	50.20 ± 10.44 ^a^	5.63 ± 1.36 ^a^	19.16 ± 11.02 ^a^
+Simvastatin	30.40 ± 1.90 ^a^	39.70 ± 3.28 ^a,c^	4.51 ± 0.93 ^a^	9.33 ± 2.82 ^b,c^
+Silymarin	30.24 ± 0.94 ^a^	44.61 ± 7.88 ^a^	5.38 ± 1.14 ^a^	14.38 ± 7.53 ^a^
+Metformin	30.41 ± 1.71 ^a^	39.31 ± 4.81 ^ac^	5.09 ± 1.25 ^a^	8.90 ± 4.36 ^c^
+RG 250 mg/kg	30.29 ± 1.22 ^a^	44.54 ± 9.23 ^a^	4.54 ± 0.76 ^a^	14.25 ± 8.33 ^b^
+fRG 500 mg/kg	30.19 ± 1.19 ^a^	39.28 ± 9.48	4.84 ± 0.86 ^a^	9.09 ± 9.27
+fRG 250 mg/kg	30.30 ± 1.11 ^a^	44.31 ± 5.81 ^a^	4.58 ± 1.16 ^a^	14.01 ± 5.66 ^a^
+fRG 125 mg/kg	30.58 ± 1.29 ^a^	42.56 ± 7.83 ^a^	4.94 ± 1.00 ^a^	11.99 ± 8.86

Values were expressed mean ± S.D. of eight mice (Significant as compared with ND-fed mice, ^a^ *p* < 0.01; ^b^ *p* < 0.05; significant as compared with HFD-fed mice; ^c^ *p* < 0.05; RG, Red Ginseng; fRG, Red-koji fermented RG; HFD, high fat diet).

**Table 2:** Organ weights after 84 days of continuous oral treatment of fRG in HFD-fed mice.

Groups	Periovarian fat		Liver		Kidney	
	Absolute (g)	Relative (% of body weights)	Absolute (g)	Relative (% of body weights)	Absolute (g)	Relative (% of body weights)
ND	0.072 ± 0.030	0.219 ± 0.091	1.315 ± 0.165	3.973 ± 0.291	0.200 ± 0.019	0.605 ± 0.028
HFD	0.530 ± 0.155 ^a^	1.059 ± 0.252 ^a^	1.572 ± 0.202 ^a^	3.182 ± 0.334 ^a^	0.237 ± 0.025 ^a^	0.486 ± 0.084 ^a^
+Simvastatin	0.189 ± 0.129 ^b,c^	0.464 ± 0.299 ^b,c^	1.266 ± 0.123 ^c^	3.198 ± 0.366 ^a^	0.223 ± 0.030	0.563 ± 0.076
+Silymarin	0.441 ± 0.291 ^a^	0.918 ± 0.508 ^a^	1.423 ± 0.174	3.259 ± 0.589 ^a^	0.220 ± 0.026	0.506 ± 0.111 ^b^
+Metformin	0.179 ± 0.104 ^b,c^	0.446 ± 0.256 ^b,c^	1.527 ± 0.146 ^b^	3.910 ± 0.402 ^c^	0.237 ± 0.031 ^a^	0.604 ± 0.051 ^c^
+RG 250 mg/kg	0.412 ± 0.251 ^b^	0.864 ± 0.463 ^b^	1.406 ± 0.156	3.226 ± 0.428 ^a^	0.249 ± 0.030 ^a^	0.517 ± 0.101 ^b^
+fRG 500 mg/kg	0.289 ± 0.234 ^d^	0.695 ± 0.507 ^b^	1.449 ± 0.116	3.869 ± 0.903	0.224 ± 0.016	0.602 ± 0.158
+fRG 250 mg/kg	0.308 ± 0.099 ^a,c^	0.691 ± 0.198 ^a,c^	1.435 ± 0.241	3.237 ± 0.293 ^a^	0.209 ± 0.030 ^d^	0.474 ± 0.061 ^a^
+fRG 125 mg/kg	0.308 ± 0.278	0.685 ± 0.605	1.389 ± 0.184 ^b^	3.360 ± 0.734	0.202 ± 0.032 ^d^	0.481 ± 0.066 ^a^

Values were expressed mean ± S.D. of eight mice (Significant as compared with ND-fed mice, ^a^ *p* < 0.01; ^b^ *p* < 0.05; significant as compared with HFD-fed mice; ^c^ *p <* 0.01; ^d^ *p* < 0.05).

### 3.2. Effect of fRG on HFD-Induced Hyperglycemia

**Figure 3:**
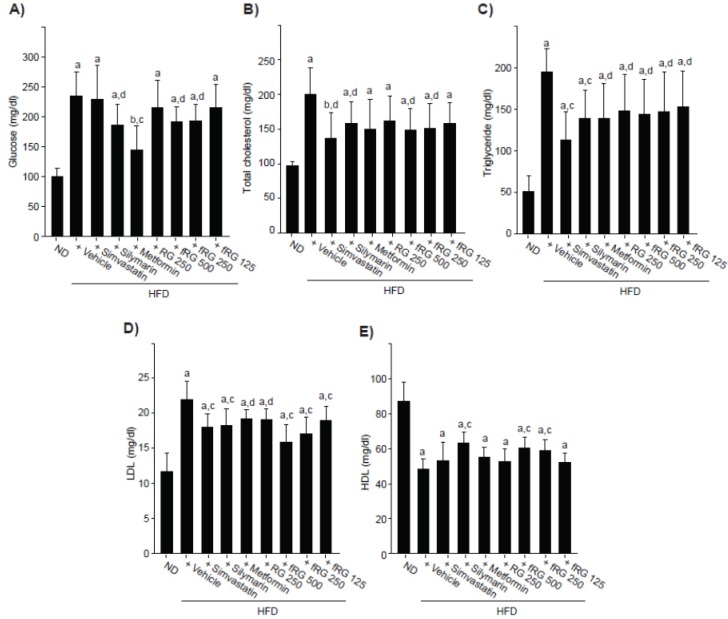
Effect of fRG on the level of serum glucose and lipid profile. The glucose (**A**), cholesterol (**B**); triglyceride (**C**); LDL (**D**); and HDL (**E**) levels were measured in the blood of ND-, HFD-, HFD + simvastatin-, HFD + silymarin-, HFD + metformin-, HFD + RG- and HFD + fRG-fed mice. All values were expressed mean ± S.D. of eight mice (Significant as compared with ND-fed mice, ^a^ *p* < 0.01; ^b^ *p* < 0.05; significant as compared with HFD-fed mice, ^c^ *p* < 0.01; ^d^ *p* < 0.05).

The blood glucose level was significantly increased by HFD administration. Glucose levels in HFD control changed by 133.66% compared with ND-fed mice and by −2.55%, −20.52%, −38.38%, −8.35%, −18.18%, −17.76% and −8.51% in simvastatin, silymarin, metformin, RG, fRG 500, 250 and 125 mg/kg treated mice compared with HFD-fed mice, respectively. Although RG did not inhibit the HFD-mediated increase in blood glucose, 500 and 250 mg/kg fRG treatments significantly alleviated the HFD-mediated increase in blood glucose (Figure 3A).

### 3.3. Effect of fRG on HFD-Induced Hyperlipidemia

Significant increases of serum total cholesterol (Figure 3B), triglyceride (Figure 3C), and LDL (Figure 3D) were observed in HFD-fed mice. Although RG administration did not reduce the HFD-induced level of total cholesterol, fRG treatment (500 and 250 mg/kg) significantly decreased the total cholesterol induced by HFD (Figure 3B). In addition, all dosages of fRG administration significantly inhibited the HFD-increased levels of triglyceride and LDL (Figure 3C,D). fRG (250 mg/kg) decreased serum cholesterol and LDL more favorably but similarly inhibited triglyceride compared with an equal dose of RG. Moreover, HDL was significantly reduced in HFD-fed mice compared to ND-fed mice, and those reductions were blocked only by 500 and 250 mg/kg fRG treatments (Figure 3E).

### 3.4. Effect of fRG on HFD-Induced Hepatopathy

**Figure 4:**
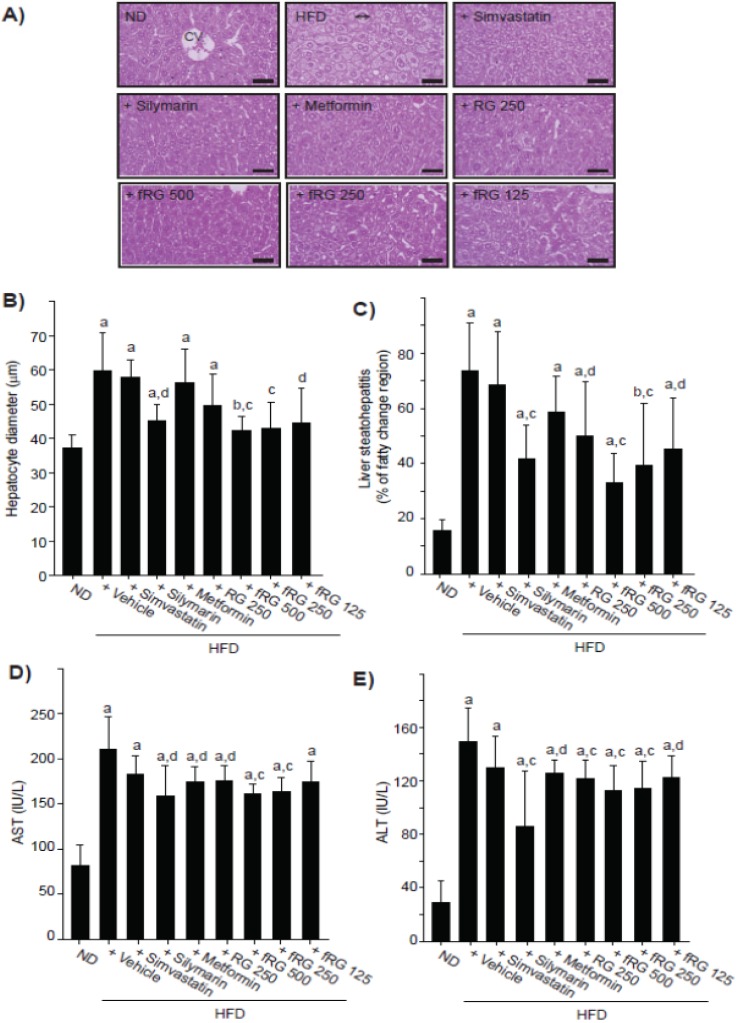
Effect of fRG on HFD-induced liver damages. The representative histological images of liver tissues (**A**) were stained with H & E (Scale bars = 80 μm). The mean diameter at least 10 hepatocytes was measured using automated image analysis process (**B**). Percentage of fatty changed regions (liver steatohepatitis) was calculated in 1 mm^2^ of hepatic parenchyma (**C**). The levels of AST (**D**) and ALT (**E**) were also measured as described in Figure 3. All values were expressed mean ± S.D. of eight mice (Significant as compared with ND-fed mice, ^a^ *p* < 0.01; ^b^ *p* < 0.05; significant as compared with HFD-fed mice, ^c^ *p* < 0.01; ^d^ *p* < 0.05).

HFD-fed mice exhibited increased absolute liver weight and decreased relative weight, and those changes were not altered by fRG administration (Table 2). Histomorphometrical analyses indicated that HFD increased mean hepatocyte diameter (hepatocyte hypertrophy; Figure 4A,B) and steatohepatitis (percentage of fatty changed regions in liver parenchyma; Figure 4A,C). Although RG administration did not reduce mean hepatocyte diameter, all dosages of fRG treatment significantly inhibited the hypertrophy of hepatocyte (Figure 4B). In addition, HFD-mediated steatohepatitis was significantly alleviated by fRG in a dose-dependent manner (Figure 4C). We next monitored the levels of AST (Figure 4D) and ALT (Figure 4E) as blood markers for hepatopathy. All dosages of fRG treatment inhibited the increased levels of AST and ALT by HFD. More favorable inhibitions of HFD-mediated hepatopathy were observed with fRG treatment than with an equal dose of RG.

### 3.5. Effect of fRG on HFD-Induced Nephropathy

**Figure 5:**
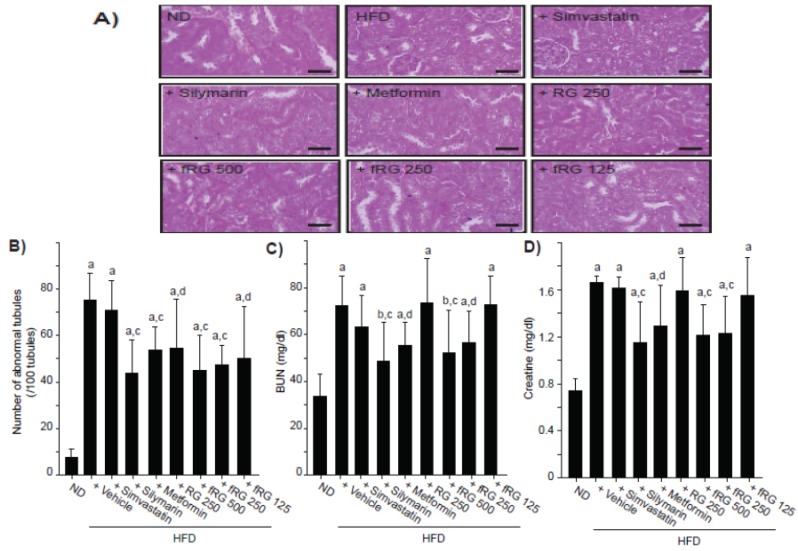
Effect of fRG on HFD-induced kidney damages. The representative histological images of kidney (**A**) were stained with H & E (Scale bars = 80 μm). The number of vacuolated tubules among 100 tubules (**B**), BUN (**C**), and creatinine (**D**) in blood were measured in ND-, HFD-, HFD + simvastatin-, HFD + silymarin-, HFD + metformin-, HFD + RG- and HFD + fRG-fed mice. All values were expressed mean ± S.D. of eight mice (Significant as compared with ND-fed mice, ^a^ *p* < 0.01; ^b^ *p* < 0.05; significant as compared with HFD-fed mice; ^c^ *p* < 0.01; ^d^ *p* < 0.05).

Similar to liver weights, absolute and relative kidney weights were also increased and decreased, respectively, and those changes were not altered by fRG treatment (Table 2). Histomorphometrical analyses showed that HFD increased the number of abnormal kidney tubules by vacuolation (Figure 5A,B). However, abnormal tubules were significantly reduced by all dosages of fRG (Figure 5B). Furthermore, 250 mg/kg fRG more favorably inhibited the number of abnormal tubules compared with an equal dose of RG. Finally, we monitored the level of BUN (Figure 5C) and creatinine (Figure 5D) as blood markers for nephropathy. fRG treatment (500 and 250 mg/kg), but not RG treatment, significantly decreased the HFD-induced level of BUN and creatinine. 

## 4. Discussion

Ginseng has been widely used as an herbal remedy for various disorders by enhancing body strength, recovering physical balance, and stimulating metabolic function [13]. Because RG restores and enhances normal well-being, RG made by steaming and subsequently drying *P*. *ginseng* has been regarded as an adaptogenic [14,20]. RG ameliorates metabolic syndrome by improving the insulin sensitivity of peripheral tissue, and enhancing the serum lipid profile, inhibiting obesity, and so on [10,15,21]. Several molecular mechanisms to alleviate metabolic syndrome have been extensively studied, and those might be regarded as the putative target of RG. Among them, protopanaxadiol-type ginsenoside and compound K decrease hepatic gluconeogenesis through the inhibition of phosphoenolpyruvate kinase and glucose-6-phosphatase expression [22]. It has also been reported that glucagon-like peptide 1, which is a potent anti-diabetic hormone to stimulate insulin secretion, is increased by total saponin and ginsenoside Rb1 administration in HFD/streptozotocin-treated animals [23]. In addition, several lines of evidence indicate that adenosine monophosphate-activated protein kinase, which is also activated by ginseng and its active components [24,25,26,27], promote glucose uptake and mitochondrial biogenesis in muscle [25,26], inhibit hepatic gluconeogenesis [27], and prevent adipogenesis and inflammation [28].

Interestingly, the pharmacological effects of RG were further increased by fermentation. It has been reported that fermentation with *Phellinus linteus*, *Bifidus*, or *Lactobacillus fermentum* shifts the chemical composition of ginsenosides and improves antioxidant, hypolipidemic, hypoglycemic and anti-inflammatory activities [16,17,18,29,30,31]. On the other hand, Red-koji from *Monascus* species has been used as a medicinal food and fermentation source for other herbs in East Asia including China, Korea and Japan [19]. Our preliminary UPLC analysis also showed that the chemical composition of the major ginsenosides was changed and new peaks were detected in red-koji-fermented RG (data not shown). Therefore, active compounds of fRG and their pharmacological relevance need to be further clarified in the near future.

In the present study, we investigated anti-obesity, hypoglycemic, hypolipidemic, hepatoprotective and nephroprotective effects of three different dosages of fRG in HFD-fed mice for 84 days, and the efficacies were compared to those of non-fermented red ginseng, simvastatin (for hypolipidemic activity), silymarin (for hepatoprotective and free radical scavenger effects) and metformin (for hypoglycemic and related anti-obesity effects). The HFD-induced animal model is a well-established model of induced obesity, and the obese mice also have the characteristics of metabolic disorders including hyperglycemia, insulin resistance, hepatic steatosis, hyperlipidemia and obesity-mediated nephropathy [5,9,10,11]. As expected, the present results showed marked obese states (increased body weight and fat depositions, adipocyte hypertrophy), hyperglycemia, hyperlipidemia (increased serum total cholesterol, triglyceride and LDL levels with decreased HDL levels), liver (increased serum AST and ALT level with severe steatohepatitis) and kidney damages (fatty changes in tubules, serum BUN and creatinine elevations) by 91 days of continuous HFD supply.

The accumulation or increase of fat deposition in the body is a major characteristic of obesity, and cellular hypertrophy appears to be the major mode of expansion of the intra-abdominal adipose tissue in rodents [32]. The present results indicate that HFD increases body and fat weights, as well as the thickness of abdominal adipose tissues with severe hypertrophy of adipocytes. Although we did not observe significant changes in body weight gains with fRG in HFD-fed mice, fRG treatment tended to decrease body weight gain in a dose-dependent manner (Figure 1A and Table 1). In addition, fRG (500 and 250 mg/kg) significantly reduced periovarian fat accumulation, while RG did not prevent the accumulation (Table 2). Moreover, all dosages of fRG effectively inhibited the fat histopathological changes by HFD supply (Figure 2C,D). More favorable anti-obesity effects were observed with fRG than with an equal dose of RG. Taken together, the present results provide direct evidence that fRG has anti-obesity activity, and the effects of RG were increased by fermentation with Red-koji. We also found that a decrease of mean daily food consumption in HFD-fed mice did not change in any tested group. Thus, the anti-obesity effects of fGR were not from the inhibition of food consumption.

HFD-fed mice have been used as an animal model for type II diabetes [10,11]. In the present study, a more favorable reduction of glucose levels was observed with fRG compared to an equal dose of RG. On the other hand, the efficacy of hypolipidemic agents is generally evaluated based on the decrease of serum LDL, triglyceride and total cholesterol with increased HDL levels [33,34]. In the present results, HFD-induced hyperlipidemia was markedly inhibited by treatment with fRG 500 and 250 mg/kg, which was confirmed by decreases in serum LDL, total cholesterol and triglyceride and the increase in HDL. In addition, more favorable hypolipidemic effects were observed with fRG as compared with an equal dose of RG. Therefore, the hypoglycemic and hypolipidemic effects of RG were increased by fermentation with red-koji.

Generally, hypertrophy and fatty change of hepatocytes are accompanied by increased AST and ALT activities in HFD-fed mice [35,36]. In addition, chronic metabolic disorders involving diabetes increase the risk of inflammation and fatty changes in kidney, and thereby elevate the level of BUN and creatinine by renal dysfunction [37]. The present results clearly showed that all measured histological and biochemical parameters related to steatohepatitis, except the AST level in the 125 mg/kg fRG-treated group, were effectively decreased by three different dosages of fRG treatment. Similar to the hepatoprotective effect of fRG, 84 days of continuous treatment with fRG (500 and 250 mg/kg) decreased the number of fatty tubules and the serum BUN and creatinine levels. More favorable hepatoprotective and nephroprotective effects were observed with fRG than with an equal dose of RG.

## 5. Conclusions

In conclusion, the results obtained in the present study suggest that 84 days of continuous oral treatment with fRG effectively ameliorated HFD-induced metabolic disorders involving abnormal fat disposition, hyperglycemia, hyperlipidemia, hepatopathy, and nephropathy. The overall suitable effective dose of fRG for HFD-mediated metabolic complications was considered to be about 250 mg/kg/day in mice. Therefore, the present study provides direct evidence that the pharmacological activities of RG were enhanced by red-koji fermentation, and fRG could be a neutraceutical resource for the alleviation of obesity-mediated metabolic disorders.
